# Implementation of a registry and open access genetic testing program for inherited retinal diseases within a non‐profit foundation

**DOI:** 10.1002/ajmg.c.31825

**Published:** 2020-08-11

**Authors:** Brian C. Mansfield, Benjamin R. Yerxa, Kari H. Branham

**Affiliations:** ^1^ Foundation Fighting Blindness Columbia Maryland USA; ^2^ Kellogg Eye Center, University of Michigan Ann Arbor Michigan USA

**Keywords:** clinical consortium, inherited retinal diseases, open access genetic testing, patient registry, venture philanthropy

## Abstract

The Foundation Fighting Blindness is a 50‐year old 501c(3) non‐profit organization dedicated to supporting the development of treatments and cures for people affected by the inherited retinal diseases (IRD), a group of clinical diagnoses that include orphan diseases such as retinitis pigmentosa, Usher syndrome, and Stargardt disease, among others. Over $760 M has been raised and invested in preclinical and clinical research and resources. Key resources include a multi‐national clinical consortium, an international patient registry with over 15,700 members that is expanding rapidly, and an open access genetic testing program that provides no cost comprehensive genetic testing to people clinically diagnosed with an IRD living in the United States. These programs are described with particular focus on the challenges and outcomes of establishing the registry and genetic testing program.

## 1 INHERITED RETINAL DISEASES

The inherited retinal diseases (IRD) are a group of rare genetic diseases that affect the neural retina of the eye limited to members of the Registry who lived in the United States and often lead to a progressive loss of vision that may result in blindness. Within the United States it is estimated there are 200,000–300,000 people affected by an IRD, which projects a worldwide prevalence estimate of 4.5–6.8 million people (Daiger, Bowne, & Sullivan, 2007; Daiger, Sullivan, & Bowne, 2013). A recent analysis of just the autosomal recessive (AR) IRD reported a genetic prevalence of 1 case in 1,380 individuals, with 5.5 million people predicted worldwide (Hanany, Rivolta, & Sharon, 2020) with 2.7 billion people worldwide (36% of the population) healthy carriers of at least one mutation that can cause AR‐IRD, possibly among the highest across any group of human Mendelian diseases. Each of the IRD are orphan diseases. The majority of the diseases are monogenic and over 270 genes have so far been implicated (RetNet, https://sph.uth.edu/RetNet/) (Daiger, Rossiter, Greenberg, Christoffels, & Hide, 1998), accounting for 55–60% of the disease burden (Bujakowska et al., 2016; Haer‐Wigman et al., 2017; Zampaglione et al., 2020). Clinically the diseases can be diagnosed in three broad categories, those that affect the central retina initially and increase peripherally over time; those that affect the periphery first, then spread centrally, and those that are congenital and stationary. Within each of those categories there is great diversity in age of onset, rate of progression, and mode of inheritance (Sahel, Marazova, & Audo, 2015). Traditional clinical diagnosis has been based on named disease nomenclature representing the initial clinical presentation, such as Leber congenital amaurosis, for early childhood onset disease, retinitis pigmentosa (RP) for diseases starting peripherally and moving centrally and Usher syndrome, for diseases that involved hearing loss in addition to vision loss. With the increased knowledge about the genetic cause of disease, however, there has been a greater focus on a gene‐specific disease nomenclature. For instance, pathogenic variants in the gene *USH2A*, while initially identified as the cause of Usher syndrome type 2A, are now known to be the most common cause of disease in AR non‐syndromic RP (Pontikos et al., 2020). Similarly, the genes *CRX* and *PRPH2* are each implicated in at least three different retinal diseases—Leber congenital amaurosis, RP, and cone/cone‐rod dystrophies (Leroy, Pennesi, & Ohnsman, 2018). For most of the diseases, there is no clear genotype–phenotype relationship (Cremers, Boon, Bujakowska, & Zeitz, 2018).

Prior to the 2018 approval of the gene augmentation therapy Luxturna® (voretigene neparvovec) by the FDA for retinal disease caused by biallelic pathogenic variants in the *RPE65* gene there were no approved therapies for any IRD. While still the only FDA approved therapy, there is now a vigorous pipeline of clinical trials with promising therapies, due in large part to a 50‐year history of investment and advocacy by people affected by IRD in the Foundation Fighting Blindness.

## 2 THE FOUNDATION FIGHTING BLINDNESS

Earnest research into ocular diseases started in 1968 with the establishment of the National Eye Institute (NEI). In 1971, to increase awareness and research into the rare IRD RP, a group of affected families, led by the Berman and Gund families, formed the National Retinitis Pigmentosa Foundation. In 1974, the Foundation established one of the first dedicated research laboratories in the United States, the Berman‐Gund Laboratory led by Dr Eliot Berson at Massachusetts Eye and Ear at Harvard Medical School, which in 1990 described the first genetic basis of RP (Dryja et al., 1990) and initiated the near exponential increase in IRD gene discovery. As the increasing genetic diversity and overlap between RP and other IRD grew, the Foundation was renamed to the Foundation Fighting Blindness. The Foundation continues to invest ~25% of its funds in gene discovery and characterization, supporting increasingly sophisticated genetic tools to discover the genetic cause of the remaining 40–45% of unsolved IRD cases (Bronstein et al., 2020), and supports a nationwide Israeli IRD consortium performing clinical and genetic mapping of the entire Israeli IRD population (Sharon et al., 2020). In total the Foundation has raised over $760 M for IRD research with an annual research budget of over $20 M that supports over 73 investigators across 14 countries.

The current mission of the Foundation is to support the development of treatments and cures for the inherited retinal dystrophies and age‐related macular degeneration where there are clear genetic drivers of disease. To achieve this goal, the programs of the Foundation cover a broad spectrum (Shaberman & Durham, 2019) and include: clinical career development awards for young and established investigators; individual and multiple investigator preclinical and clinical research awards; mentored translational research acceleration awards pairing experienced industry drug developers with promising academic research (https://www.fightingblindness.org/grants-and-award-programs); non‐rodent animal model awards to support the development of new genetic models with larger eyes; and a canine IRD facility (Beltran, 2009) co‐funded with the NEI to accelerate bench to bedside research in a large clinically relevant eye (https://www.vet.upenn.edu/research/centers‐laboratories/research‐laboratory/experimental‐retinal‐therapies/publications#2001). Funding decisions and strategic directions for preclinical and clinical research are guided by a scientific advisory board of 54 international leading researchers and clinicians in IRD (https://www.fightingblindness.org/about/scientific-advisory-board).

## 3 CLINICAL CONSORTIUM

Accurate diagnosis, characterization and treatment of patients with IRD requires both clinical and genetic characterization of disease. In 2013 the Foundation funded an international nine center natural history study of Stargardt disease due to pathogenic variants in the *ABCA4* gene, ProgStar (NCT01977846) (Strauss et al., 2019) that resulted in over 14 publications and the identification of relevant clinical endpoints (http://progstar.org). Building on this model, in 2016 the Foundation created a clinical consortium which currently consists of over 38 IRD centers of excellence across 11 different countries (https://public.jaeb.org/ffb/clin). The goal of the consortium is to accelerate clinical translation of promising therapies by undertaking robust, high‐quality, multi‐center clinical studies that are shared openly. Studies generate data using standardized protocols, a central coordinating center (JAEB) and study‐certified reading centers. De‐identified data from the completed trials are archived in an open central repository to stimulate further hypothesis generation and innovation. Currently a natural history study of diseases caused by pathogenic variants in the *USH2A* gene, called RUSH2A (NCT03146078) is following 127 patients over 4 years is in progress (Duncan et al., 2020) and a second study on people with pathogenic variants in the *EYS* gene called Rate of Progression *in EYS* Related Retinal Degeneration (Pro‐EYS) (NCT04127006), a cause of AR RP, is also in progress.

## 4 THE RD FUND

Historically the Foundation has raised funds through traditional community‐based nonprofit approaches which it invested in awards to investigators that range from $30,000 to $500,000 per year. However, to accelerate the pace of clinical progress in moving from laboratory drug development to approved clinical products, costs are tens, if not hundreds, of millions of dollars for each program, and have a very high failure rate. To meet this challenge requires innovation in funding models, such as leveraging investments to attract outside venture capital. To accomplish this, the Foundation launched the Retinal Degeneration Fund (RD Fund) in 2018 (https://www.retinaldegenerationfund.org/) as a 501(c)(3) not‐for‐profit venture philanthropy organization. With over $70 million of capital to invest, the Fund focuses on making mission‐related investments, preferably for programs within 18 months of initiating clinical proof of concept studies, with any returns reinvested in the Foundation. Currently the portfolio contains eight companies with investments ranging from $250 K up to $7.5 M.

## 5 MY RETINA TRACKER REGISTRY

One challenge for rare genetic diseases is identifying the affected population. Few eyecare professionals see a case of an IRD or can provide a clear diagnosis. A study by Achroma Corp commissioned by the company AGTC in 2018 showed that for adults with achromatopsia, the patient journey took over 5 years and on average seven different healthcare providers for a diagnosis. Notably only 58% of adults and 65% of children with achromatopsia received genetic testing to support the clinical diagnosis (Achroma Corp, 2018). Many people affected with an IRD do not complete the diagnostic journey but instead seek practical support at low vision centers. This creates barriers to accelerating treatments and cures. It also impacts our understanding of the true prevalence, clinical diversity, geographic distribution, age and rate of progression of disease in the population, and the ease of enrolling eligible patients into research and clinical studies. Upon commercialization of a therapy this lack of information about these conditions slows the speed of market penetration, which are key considerations for investors financing drug development. A registry easily accessible to people affected by the IRD can help address these issues.

**FIGURE 1:**
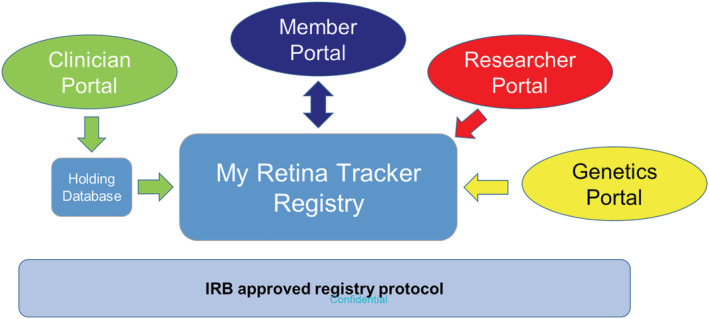
Structure of the My Retina Tracker Registry. People affected with an inherited retinal disease (IRD) join the Registry through a Member Portal https://www.fightingblindness.org/my‐retina‐tracker‐registry Following an online informed consent, members are presented with surveys to capture their objective experience of living with an IRD. During a visit to a clinician, the member can request the clinician enter the clinical ophthalmic exam results through a Clinician Portal on the same web site. To simplify use, clinical data entry is one way, requires no prior authorization, username or password, and initially enters a holding database. Clinical data is released from the holding database into the members profile once an algorithm run by the Registry Coordinator identifies a matching profile in the Registry database. Genetic testing data generated by the CLIA‐certified genetic testing partner lab can be downloaded electronically from the lab directly into the registry and matched to the correct member profile. Both the pdf genetic report and the complete set of sequence variants detected are transferred into the database along with their classification. Researchers, approved for access, can view and download de‐identified data either through a dedicated researcher portal or in collaboration with the Registry staff who may perform searches on their behalf

The Foundation had maintained a patient registry for many years, that grew to 11,000 names, but was little more than a contact list of patients with IRD, but had limited disease information. In 2014, to improve data quality and depth a more detailed on‐line registry was launched, under an Institutional Review Board (IRB) approved protocol, branded My Retina Tracker® Registry https://www.fightingblindness.org/my-retina-tracker-registry (Fisher, Bromley, & Mansfield, 2016). The goals of the Registry are to provide a single, integrated source of information about, and connection to, all people with an IRD; and to share those data, de‐identified, with researchers and partners, in order to accelerate the development of treatments and cures. The Registry provides a convenient, secure database to aggregate information about people affected with an IRD (Figure 1). Membership is initiated when an affected person chooses to join and provides online informed consent to share de‐identified data, and be contacted by Registry staff if there is an opportunity, they may be interested in. Members own and control their own data. Once consented, members complete a series of short surveys to capture their subjective experience of living with their retinal disease, information about their health history, how they adjust their life around their disease, family history, and genetic cause of disease. During a clinical consult, members can ask their clinician to enter the objective clinical measurements through a clinical portal (Figure 1). A research portal enables data analysis of all Registry de‐identified data for approved, external researchers. Members are encouraged to update their personal surveys at least once a year and the longitudinal data provides a perspective on disease progression. The member and clinician surveys use a controlled vocabulary primarily in the form of standardized dropdowns for answers, to facilitate efficient data mining.

In 2020, the Registry underwent a major upgrade. Key upgrades included enhanced security features; global compliance with data privacy rules, including GDPR and U.S. data and patient protection laws; and mobile‐SMS integration to facilitate new and existing surveys and provide a more interactive platform. The validated Patient‐Reported Outcomes Measurement Information System® 29 question survey (PROMIS‐29), a tool designed to measure self‐reported physical, mental and social health and wellbeing (Cella et al., 2019) and determine quality adjusted life years (QALYs) (Craig et al., 2014) was implemented. Other validated patient reported outcomes (PRO) and outcomes research instruments are planned.

Since launch, over 15,700 people have created an online Registry profile. The baseline growth of the Registry is ~100 new members per month, but with the introduction of no‐cost genetic testing, that growth has averaged over 370 per month and continues to increase. The Registry also houses the contact information for the 11,000 registrants from the earlier registry, although many of those have failed to re‐engage, possibly representing the age and history of the information.

The total 26,700 Registry membership is 48% male, 45% female with the remainder choosing not to declare their sex and the average age 50.2 years (±20.6 1*SD*). For the 15,700 actively engaged members who have created a profile since the Registry went online, the membership is 44% male, 43% female with the remainder choosing not to declare their sex and an average age of 44.3 years (±20.7 1*SD*). These differences in the two membership groups align with the history of the Registry. While most enrollees reside in the United States (94%), 112 countries are represented, with 18 countries representing 75% of the international membership. The most represented in international membership are: Canada (14%), United Kingdom (8.9%), India (8.2%), Italy (7.1%), Mexico (6.5%), South Africa (5%), Australia (4.5%), Poland (4.2%), Germany (3.5%), Argentina (2.2%), Brazil (1.8%), France (1.7%), Netherlands (1.6%) and New Zealand (1.3%). Of the international members, 97% have joined recently with an online profile, the members from the earlier registry being predominantly from Canada.

**FIGURE 2:**
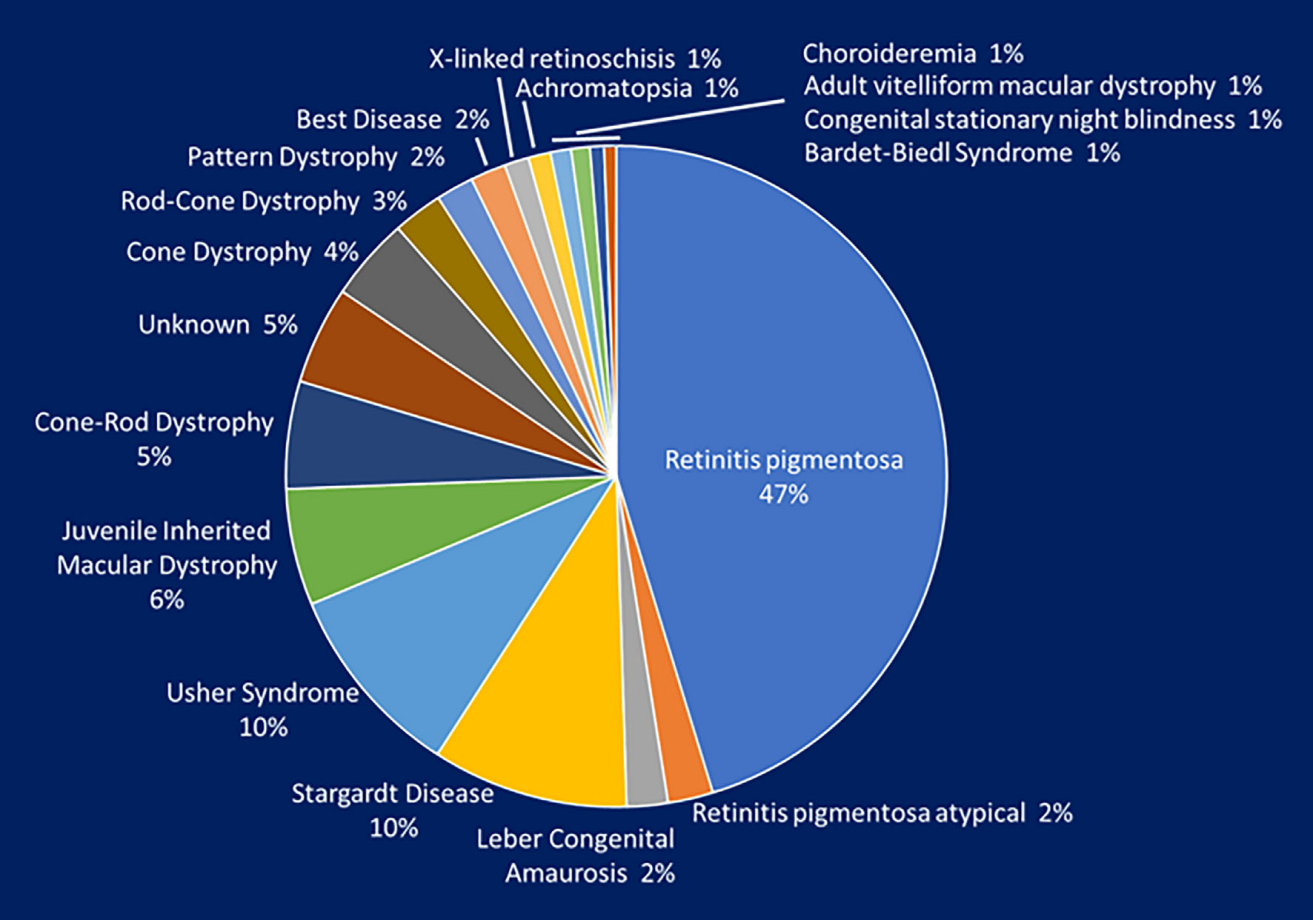
Composition of the My Retina Tracker Registry. The composition of the current Registry membership, by clinical diagnosis, for the 15,700 members with an online profile

The composition of the current Registry data by clinical diagnosis is shown in Figure 2. RP, including Leber congenital amaurosis, accounts for 51% of the members' diagnoses. Stargardt disease and all forms of Usher syndrome account for 10% each followed by juvenile inherited macular dystrophy at 6%. Currently 5% of cases are clinically characterized as unknown.

Data in the Registry is currently accessible via the Registry staff. Non‐profit use is supported at no cost, while for‐profit users sign a consulting contract to help offset the costs of Registry operation. De‐identified data, lacking names, contact information or demographics below state/province level can be requested. If researchers are interested in contacting Registry participants, an IRB approved contact letter must be submitted to Registry staff outlining the identity of the interested party, their reason for contacting the Registry member, and contact information for the member to use if they wish to pursue the opportunity. Once approved, Registry staff send a contact letter to the selected Registry members. The decision to identify themselves or not rests entirely with the member, and subsequent interactions with the interested party are independent of the Registry.

External interest in the Registry grew rapidly. There have been over 44 substantial requests for data including requests to: help enroll in nine clinical trials, multiple natural history studies and multiple focus groups; provide prevalence for specific genes, variants and technology‐specific attributes; provide DNA for preclinical research; promote IRD disease specific conferences; and support a Retina International survey on the economic impact of blindness.

## 6 REMOVING THE ACCESS BARRIER FOR GENETIC TESTING

The current preclinical and clinical pipelines for the IRD are heavily weighted toward gene and variant specific diseases. The first FDA approved in vivo gene augmentation therapy is specific to the *RPE65* gene, and there is a pipeline of 15 different gene augmentation trials for the IRD in over 24 different clinical trials. Similarly, the first human in vivo CRISPR/Cas9 gene editing clinical trial, sponsored by Editas Medicine and Allergan is for a specific variant in intron 26 (c.2991+1655AG, p.Cys998X) of the *CEP290* gene, as are antisense oligonucleotide‐based variant specific trials by ProQR for the *USH2A* gene exon 13 mutation (c.2299delG, p.Glu767Serfs*21), *RHO* gene (*c*.68C>A, p.Pro23His), and *CEP290* gene (c.2991+1655AG, p.Cys998X) variants. The genetic cause has become a critical component augmenting a clinical diagnosis. Prior to 2017 ~10% of My Retina Tracker Registry members reported having a genetic test.

In January 2017 the Foundation launched a pilot program to understand the patient and clinical interest in genetic testing by funding a comprehensive IRD genetic testing and counseling service at no cost to patient, clinician or insurance. The program was limited to people living in the United States and designed to address the problems faced within the United States for access to testing. Models seeking to minimize the cost using patient insurance were considered, but reimbursement rules, and the Foundation acting essentially as a co‐insurer, created administrative complications and would specifically exclude Medicaid patients who only receive last resort coverage.

To reduce the Foundations administrative workload, and provide a consistent dataset for later analysis, a single genetic testing provider was selected. Key considerations were for a comprehensive IRD gene panel test with strong coverage of the genetic regions known to be difficult, such as the 1kb long purine rich region of ORF15 within the *RPGR* gene (Vervoort et al., 2000; Vervoort & Wright, 2002) which is reported to account for 80% of all cases of *RPGR* mutations, sensitivity for the increasing number of deep intronic pathogenic variants being discovered in genes like *ABCA4* (Sangermano et al., 2019), and high sensitivity copy number detection, since these variants may represent 9% of IRD cases (Zampaglione et al., 2020).

Turn‐around time was also important. In the past, Registry members had sought Registry staff help to obtain results for genetic testing they had participated in many years prior. Expecting results in weeks, members expressed frustration and lack of confidence in testing when there had been no communication of results after a year, often more, and their enquiries not returned. In most cases the member had participated in an academic research study, which had failed to identify a genetic cause. Communicating the difference between research studies and a CLIA‐certified test, and education that a CLIA‐certified test would provide a prompt result, even if negative, was important. Given the complexity of a genetic result, and the enquiries we had previously from constituents who had been tested, but results had not been explained to them, genetic counseling was considered an essential aspect for our program. Genetic counseling was provided through genetic counselors associated with IRD centers when available, or otherwise provided by InformedDNA telegenetic counselors who could support patients nationwide. Blueprint Genetics was selected as the genetic testing lab.

The pilot program was an IRB approved protocol within the Registry Protocol. Eligibility was limited to members of the Registry who lived in the United States, who completed an informed consent, had not previously had a relevant comprehensive gene panel test, and agreed to upload the result into their de‐identified Registry profile. To order the test a clinician was required to enter, at minimum, a clinical diagnosis of an IRD and a recent best corrected visual acuity (BCVA) into the Registry clinical portal. Registry staff confirmed all eligibility criteria before approving Blueprint Genetics to test and invoice the Foundation. During genetic counseling, the test result was entered into the Registry clinical portal, before invoicing the Foundation. Initially 10 clinicians with a strong IRD expertise were approved to order the test. Demand from patients and clinicians to expand the program led, over 22 months, to over 180 approved clinicians across 149 geographically diverse practice groups, of which 40% were academic and 60% private, ordering over 6,300 tests. An analysis by InformedDNA of referral data from two of the clinics with the highest referral rates, showed that prior to the program 75% of patients referred for testing reported they did not complete pre‐test genetic counseling appointments or obtain genetic testing, primarily because of lack of insurance coverage and/or cost, with genetic counseling, or testing, or both. The program reversed the trend with >98% participation of referred patients completing genetic testing through this research protocol. In 2019 a survey of the satisfaction with genetic counseling showed that 98% considered the counseling important, feeling more informed about their genetic risks and better equipped to make informed decisions about their retinal condition.

As demand from the program grew, the Foundations administrative burden ensuring eligibility and tracking invoicing became unscalable. Common challenges were patients not being in the Registry, clinicians overlooking the entry of the diagnosis and/or BCVA in the Registry and the need for ordering clinicians, especially in academic centers, to seek their IRB approval before submitting patient data into the third‐party Registry. These created significant backlogs in the testing pipeline, delaying results to patients.

To scale more efficiently an Open Access genetic testing program was launched in October 2019 using a recently expanded retinal dystrophy panel (including mitochondrial genes) of 322 genes offered by Blueprint Genetics (https://blueprintgenetics.com/tests/panels/ophthalmology/retinal-dystrophy-panel/). The key innovations were: removing the need for a patient to be a member of the Registry; capturing the required patient online consent and clinical data in a custom online ordering portal of Blueprint Genetics; offering membership in the Registry during genetic counseling; and electronic export of the patient, clinical and test result data from Blueprint Genetics into the Registry for those who are, or become, members of the Registry. Clinicians no longer needed to enter data into a third‐party Registry, removing the need for their IRB approval. Participants are informed about the Registry during genetic counseling and, if asked, the counselors assist the participant with the Registry informed consent and register them online.

Family variant testing is provided by the program for other affected family members of the proband in selected cases when informative in: determining phase for recessive disease that might support eligibility for a therapy in or near clinical trials; in strengthening variant classification; or testing other affected family members. Given the lack of genotype–phenotype correlation and potential psychological impact of a pathogenic genetic diagnosis, the Foundation has refrained from providing carrier or pre‐symptomatic testing.

As anticipated, the clinician base ordering the Open Access program grew promisingly prior to COVID‐19 closures, but also highlighted the expense of scaling. While the program has been funded primarily by generous grants from the non‐profit George Gund Foundation, and other patient advocacy groups, such as Sofia Sees Hope, a sustainable program depends on creating value for industry partners. Genetic data has multiple values to industry: to guide product development, based on market size for a gene or variant specific technology; and to aid in recruitment of patients for focus groups, natural history studies and clinical trials. More important, however, is to support rapid market penetration when a product reaches market. As mentioned previously, many IRD are relatively rare and any single IRD center of excellence may only be aware of a handful of genotyped patients, meeting eligibility criteria, to complete clinical trials. However, rare disease space market penetration requires an ability to rapidly find the majority of patients who are dispersed throughout the broader community. With little genotype–phenotype correlation, their identification depends on a broader genetic screening program, an expensive undertaking for a single industry partner, especially smaller biotech companies who pioneer the IRD therapeutic space. Using a nominal price of $1,000 for a comprehensive IRD panel genetic test, a gene accounting for 5% of RP will, on average, require 20 tests ($20,000) to find one person among those with a clinical diagnosis of RP. This cost is multiplied by the prevalence of a variant within that genetic subgroup, and several‐fold more again if eligibility criteria such as age, percent viable photoreceptors, patient interest in a specific therapy, or other are applied. Any bias in the composition of the patient population being tested further impacts cost. Given many genes, or specific genetic variants are below a 1% incidence the identification cost of a single eligible patient can rapidly become hundreds of thousands of dollars. Industry cost‐sharing a genetic testing program through a non‐profit Foundation is an attractive alternative. Industry is currently focused on a handful of the 270 IRD genes, with multiple industry partners overlapping therapeutic gene targets, but careful design can ensure all parties benefit, while also benefitting the entire IRD patient population with a genetic understanding of their disease and shared with the entire research community through My Retina Tracker Registry. The Foundation, in collaboration with Blueprint Genetics and InformedDNA is currently forging a new model to achieve this partnership model. Several early industry partners supporting the development of this program are acknowledged on the Open Access Genetic Testing Program website (https://www.fightingblindness.org/open-access-genetic-testing-program). One limitation of this program is its restriction to the United States and the need to address the unique challenges presented by the structures of the U.S. healthcare and insurance environment. In the future the Foundation is interested in exploring extension of this program to a broader international community, which may require different considerations and a different structure to address those environments.

## 7 GENETIC TESTING OUTCOMES

Currently over 8,600 of the approximately 15,700 Registry members have had a genetic test, with over 7,600 of those being provided by the Registry genetic testing programs. A breakdown of the genetic causes of disease for the first 5,879 probands tested is shown in Figure 2. One hundred and forty‐eight genes were implicated in a clear genetic diagnosis. Of these the top five genes: *ABCA4* (20%) *USH2A* (13%), *RPGR* (7%), *PRPH2* (5%) and *RHO* (5%) accounted for almost 50% of the genetic diagnoses, and the top 25 genes accounted for just over 75% of the genetic causes (Table 1). These results for the U.S. population are similar to the findings of a similarly sized U.K. IRD population study (Pontikos et al., 2020). Notable differences within the top five genes were a 1.5‐fold higher incidence of *RHO* in the U.S. population, consistent with the founder effect of the *RHO* P23H variant (Farrar et al., 1990), accompanied by a similar 2.2‐fold increased incidence of *EYS*, the most common cause of AR RP. While the incidences may be more broadly representative of the genetic incidence of IRD in the United States than single site studies (Stone et al., 2017), we anticipate more accuracy as the Open Access genetic testing program expands to a wider spectrum of referring clinicians.

**TABLE 1:** Genetic causes of disease

Number of genes	% Solved genetic cases	Genes in order of descending incidence
Top 5 genes	48.2	*ABCA4* (19.0%), *USH2A* (12.9%), *RPGR* (6.8%), *PRPH2* (4.8%), *RHO* (4.7%)
Top 10 genes	59.9	+ *EYS* (2.6%), *BEST1* (2.4%), *PRPF31* (2.4%), *CRB1* (2.3%), *RS1* (2.0%)
Top 20 genes	72.1	+ *CHM* (1.7*%)*, *BBS1* (1.7%), *RP1* (1.7%), *PROM1* (1.1%), *PDE6B* (1.1%), *CRX* (1.1%), *MYO7A* (1.0%), *NR2E3* (1.0%), *CNGA3* (1.0%), *RDH12* (0.8%)
Top 25 genes	76.0	+ *RP2* (0.8%), *CNGB3* (0.8%), *ADGVR1* (0.8%), *CERKL* (0.8%), *CEP290* (0.7%)
Top 54 genes	88.9	+ *GUCY2D* (0.7%), *SAG*, *CNGB1*, *IMPG2*, *FAM161A* (0.6%), *MERTK*, *SNRNP200*, *CACNA1F* (0.5*%)*, *RPE65*, *MAK*, *RP1L1*, *CNGA1*, *CDH23*, *IMPDH1*, *CLN3*, *PDE6A* (0.4%), *CDHR1*, *ALMS1*, *PRPF8*, *PCARE*, *GUCA1A*, *TULP1*, *NYX*, *IFT140*, *RPGRIP1*, *PRPF3*, *HK1*, *KIZ*, *CLRN1* (0.3%)

*Note*: The causative genes for the first 5,879 cases submitted to the My Retina Tracker Genetic Testing Program are provided in rank order for the cases that received a clear genetic result (pathogenic or likely pathogenic variants). The incidence of each gene () is provided for the top 25 genes with key steps in incidence indicated for the bottom 29 genes.

The overall diagnostic yield, using testing laboratory variant classifications, was 59.4% across all IRD. This was calculated for autosomal dominant disease and X‐linked disease by requiring one pathogenic or likely pathogenic variant, while for AR disease it was based on two pathogenic and/or likely pathogenic variants or the combination of a pathogenic or likely pathogenic with a variant of unknown significance. By clinical diagnosis, syndromic diseases such as Usher Syndrome and Bardet Biedl Syndrome had the highest detection rates of ~83%, while cone and cone/rod dystrophies had the lowest detection rates ~50% (Table 2). A more detailed analysis of the results is being prepared for publication.

**TABLE 2:** Diagnostic yield of genetic testing by clinical diagnosis

Clinical diagnosis	Number diagnosed	Genetically confirmed	Detection rate (%)
Usher syndrome	320	264	82.5
Other syndromic retinal dystrophy	97	81	83.5
Vitreoretinopathy	103	78	75.7
Choroidal dystrophy	93	55	59.1
Rod/rod‐cone dystrophy	3,048	1,787	58.6
Macular dystrophy	1,452	837	57.6
Cone/cone‐rod dystrophy	670	333	49.7
Overall	5,783	3,435	59.4
Other, not grouped, or unknown	96	38	39.6
Total	5,879	3,473	59.1

*Note*: The detection rate, by clinical diagnosis, for the first 5,879 cases submitted to the My Retina Tracker genetic testing programs. Other syndromic retinal dystrophies are represented by Bardet Biedl Syndrome (42 patients), Gyrate Atrophy (8 patients), Joubert syndrome (2) and various other single case or unclassified syndromes. Vitreoretinopathies were represented by Retinoschisis (91 patients), Stickler (4), FEVR (3), Unspecified Vitreoretinopathy (3), Norrie Disease (1) and Wagner Disease (1).

## 8 CONCLUSION

Non‐profit organizations, like the Foundation Fighting Blindness, can play critical roles in helping to catalyze and de‐risk drug development in rare disease spaces like the IRD by a variety of strategies that include incentivizing clinician scientists to commit to these fields, supporting early preclinical and clinical work, sponsoring natural history studies that share data widely, and leveraged investments supporting key proof of concept studies in humans. Clinical characterization of patients, supported by a comprehensive genetic testing program, and natural history studies are also critical. Through implementation of a patient Registry, the patient perspective of disease, and ease of accessibility to rare disease patients can be facilitated. Foundations can partner with other organizations and industry partners and, by removing cost barriers, ensure all people diagnosed with an inherited disease can receive an accurate genetic diagnosis.
